# The need for paradigm shift: prognostic significance and implications of standard therapy-related systemic immunosuppression in glioblastoma for immunotherapy and oncolytic virotherapy

**DOI:** 10.3389/fimmu.2024.1326757

**Published:** 2024-02-08

**Authors:** Aleksei A. Stepanenko, Anastasiia O. Sosnovtseva, Marat P. Valikhov, Anastasia A. Chernysheva, Olga V. Abramova, Victor A. Naumenko, Vladimir P. Chekhonin

**Affiliations:** ^1^ Department of Fundamental and Applied Neurobiology, V. P. Serbsky National Medical Research Center of Psychiatry and Narcology, The Ministry of Health of the Russian Federation, Moscow, Russia; ^2^ Department of Medical Nanobiotechnology, Institute of Translational Medicine, N.I. Pirogov Russian National Research Medical University, The Ministry of Health of the Russian Federation, Moscow, Russia; ^3^ Center for Precision Genome Editing and Genetic Technologies for Biomedicine, Engelhardt Institute of Molecular Biology, Russian Academy of Sciences, Moscow, Russia

**Keywords:** glioblastoma, immunotherapy, lymphopenia, oncolytic virotherapy, radiotherapy, steroid dexamethasone, temozolomide chemotherapy, total lymphocyte count

## Abstract

Despite significant advances in our knowledge regarding the genetics and molecular biology of gliomas over the past two decades and hundreds of clinical trials, no effective therapeutic approach has been identified for adult patients with newly diagnosed glioblastoma, and overall survival remains dismal. Great hopes are now placed on combination immunotherapy. In clinical trials, immunotherapeutics are generally tested after standard therapy (radiation, temozolomide, and steroid dexamethasone) or concurrently with temozolomide and/or steroids. Only a minor subset of patients with progressive/recurrent glioblastoma have benefited from immunotherapies. In this review, we comprehensively discuss standard therapy-related systemic immunosuppression and lymphopenia, their prognostic significance, and the implications for immunotherapy/oncolytic virotherapy. The effectiveness of immunotherapy and oncolytic virotherapy (viro-immunotherapy) critically depends on the activity of the host immune cells. The absolute counts, ratios, and functional states of different circulating and tumor-infiltrating immune cell subsets determine the net immune fitness of patients with cancer and may have various effects on tumor progression, therapeutic response, and survival outcomes. Although different immunosuppressive mechanisms operate in patients with glioblastoma/gliomas at presentation, the immunological competence of patients may be significantly compromised by standard therapy, exacerbating tumor-related systemic immunosuppression. Standard therapy affects diverse immune cell subsets, including dendritic, CD4+, CD8+, natural killer (NK), NKT, macrophage, neutrophil, and myeloid-derived suppressor cell (MDSC). Systemic immunosuppression and lymphopenia limit the immune system’s ability to target glioblastoma. Changes in the standard therapy are required to increase the success of immunotherapies. Steroid use, high neutrophil-to-lymphocyte ratio (NLR), and low post-treatment total lymphocyte count (TLC) are significant prognostic factors for shorter survival in patients with glioblastoma in retrospective studies; however, these clinically relevant variables are rarely reported and correlated with response and survival in immunotherapy studies (e.g., immune checkpoint inhibitors, vaccines, and oncolytic viruses). Our analysis should help in the development of a more rational clinical trial design and decision-making regarding the treatment to potentially improve the efficacy of immunotherapy or oncolytic virotherapy.

## 1 Introduction

The principal therapeutic armamentarium for patients with newly diagnosed glioblastoma (grade IV glioma) includes maximal safe resection, conventionally fractionated radiotherapy with the concurrent and adjuvant DNA-alkylating drug temozolomide, and the corticosteroid dexamethasone to treat vasogenic cerebral edema. Despite multifaceted standard therapeutic interventions, five-year overall survival remains dismal (≤7% overall by age) (1, 2).

Clinical trials of immunotherapies, including vaccines and oncolytic viruses, have demonstrated encouraging efficacy in minor subsets of patients with progressive/recurrent glioblastoma (3–8). Different patient selection criteria, recombinant viruses, protocols for vaccine preparation, and schedules and routes of vaccine or virus administration have been applied in trials. However, within individual studies, some patients exhibited significant, moderate, or no therapeutic response and infiltration of lymphocytes to the tumor site following treatment, indicating that some patients’ intrinsic factors may determine a response to therapy. Several pretreatment variables, such as age, performance status, extent of tumor resection, O-6-methylguanine-DNA methyltransferase (*MGMT)* promoter methylation status, and isocitrate dehydrogenase (IDH) mutation status, are known independent prognostic and/or stratification factors in patients with glioblastoma/gliomas (9–11). However, increasing clinical evidence suggests that additional intrinsic factors in patients, which may determine the efficacy of immunotherapeutics and correlate with overall survival, are immune-related.

The largely negative results from phase I/II, II and III clinical trials of oncolytic viruses, vaccines, and immune checkpoint inhibitors in glioblastoma are supposed to be due to a number of potential barriers to the efficacy of immunotherapeutics, including inter- and intratumoral spatial and temporal cellular genetic and phenotypic heterogeneity and plasticity, insufficient immunogenicity, neoantigenic loss under therapy pressure (e.g., epidermal growth factor receptor variant III, EGFRvIII), the low expression of major histocompatibility complex, upregulation of diverse immune checkpoint inhibitors on infiltrating T lymphocytes, and increased levels/ratios of tumor-infiltrating immunosuppressive immune subsets [e.g., macrophages, neutrophils, myeloid-derived suppressor cells (MDSCs), and regulatory T cells (Tregs)] (12–20). Although the blood-brain barrier in glioblastoma is partially disrupted (21), leading to the infiltration of innate and adaptive immune cells (20), glioblastoma is nonetheless largely characterized by the absence or exclusion of T cells in the tumor microenvironment (“cold”, “immune-desert”/”immune-excluded” phenotype) (22, 23) and T cell dysfunction, including tolerance and exhaustion (23, 24). In addition to hypoxia, the glioblastoma cell metabolism contributes to immunotherapy resistance (25). In the tumor microenvironment, anti-tumor immune cells compete with tumor cells for various nutrients (e.g., glucose, glutamine, arginine, and lipids). Increased tumor glycolysis and glutaminolysis, altered tryptophan (via the kynurenine pathway), arginine, and lipid metabolism are associated not only with limited availability of critical nutrients for immune cell functions in the tumor microenvironment but also lead to acidosis, increased accumulation of lactic acid, α-ketoglutarate, and kynurenine metabolites, negatively affecting metabolism and effector function of cytotoxic lymphocytes and promoting recruitment, differentiation, and function of immunosuppressive immune cells (25). Separately, it is worth highlighting the unique immune microenvironment of IDH-mutant gliomas that produce the oncometabolite R-2-hydroxyglutarate. These tumors are characterized by reduced infiltration of T lymphocytes, macrophages, and neutrophils, and R-2-hydroxyglutarate was shown to impair effector functions of dendritic cells, T cells, and NK cells but promote immunosuppressive phenotype of macrophages (26, 27). Finally, glioblastoma-related systemic immunosuppression involves reduced lymphocyte counts (lymphopenia), increased neutrophil, MDSCs, and Tregs counts, and defective functions of antigen-presenting, helper, and effector immune cell subsets due to altered expression of different soluble and membrane proteins (28–36).

The goal of immunotherapy is to break immunological tolerance, enhance antigen presentation, re-engage innate and adaptive immune effectors in the tumor, and establish a long-term persistent population of cytotoxic tumor-specific memory T cells. Treatment efficacy may critically depend on tumor immunogenicity and the baseline systemic immunological competence of a patient, including the counts, ratios, and functional states of different circulating and tumor-infiltrating immune cell subsets. Differentiating between immunological responders and non-responders before immunotherapy/oncolytic virotherapy is clinically relevant. It is very likely that patients with low systemic immune suppression may benefit from immunotherapy/oncolytic virotherapy much better than severely immunocompromised patients who might be non-responsive to any extent. Is the response to immunotherapy/oncolytic virotherapy correlated with the degree of lymphopenia, neutrophilia, distinct immune cell subset ratios, or other systemic and local tumor immune-related signatures or biomarkers? In clinical trials, immunotherapeutics are typically tested concurrently with, or after, standard therapy. However, standard therapy-related (iatrogenic) systemic immunosuppression, long-lasting lymphopenia, and contraction of T cell receptor repertoire diversity may negatively impact the efficacy of immunotherapy/oncolytic virotherapy. In this review, we comprehensively discuss standard therapy-promoted immunotoxicity and its implications for immunotherapy/oncolytic virotherapy clinical trials. In our accompanying review in *Frontiers in Immunology* (Systemic and local immunosuppression in glioblastoma and its prognostic significance), we focused on immunological data from patients with glioblastoma/gliomas before standard therapy, namely, tumor-related immunosuppression at baseline and the prognostic significance of different circulating and tumor-infiltrating immune cell subsets [CD4+ and CD8+ T cells, natural killer (NK) cells, neutrophils, macrophages, MDSCs, and Tregs], including neutrophil-to-lymphocyte ratio (NLR), and specifically discussed the immune landscape of *IDH*-mutant gliomas, proneural, classical, and mesenchymal molecular subtypes, as well as the features of immune surveillance of the brain.

## 2 Standard therapy-related immunosuppression in glioblastoma

### 2.1 A low post-treatment total lymphocyte count is a prognostic factor for shorter survival

Standard therapy is a major cause of immune deficiency in patients with glioblastoma, exacerbating tumor-related systemic immunosuppression. Analysis of peripheral blood before, during, and after completing chemoradiotherapy showed that standard therapy may significantly affect diverse immune cell subsets (**Table 1**) (37–42). Patients with recurrent glioblastoma have lower levels of total immune effector cells, including circulating CD3+, CD4+, and CD8+ T cell subsets, B cells, and NK cells, with reductions in naïve, central memory, and effector memory subsets, activated T cells, and proliferating Ki67+ cells, than patients with newly diagnosed glioblastoma (43). Interestingly, despite its lymphocyte-depleting effect, standard therapy (34, 37, 38, 40) or a dose-intensified temozolomide regimen (44, 45) increases the proportion of circulating Tregs.

**Table 1:** Standard therapy adversely affects different immune cell subsets.

References	Patient characteristics	Time points of blood collection	Main findings
Chiba et al. (2010) (37)	• Total: 22 • Male: 10; female: 12 • Mean age (range): 51 (19–77) • Grade III: 5; grade IV: 17	Baseline, the 6th week of RT+TMZ	Decrease in the mean numbers of lymphocytes, NK, and NKT cells; no significant change in the mean number of Tregs
Fadul et al. (2011) (38)	• Total: 25 • Male: 17; female: 8 • Median age (range): 64 (23–78) • Grade IV: 25	Baseline, 4 weeks post-RT+TMZ	Decreased absolute counts of CD3+, CD4+, CD3-CD56+ NK, CD8+CD56+ NKT, and CD19+ B cells but not CD8+ cells; no significant change in the absolute Tregs counts
Ellsworth et al. (2014) (39)	• Total: 11 • Male: 8; female: 3 • Median age (range): 63 (32–74) • Grade III: 8; grade IV: 3	Baseline, 4 and 12 weeks post-RT+TMZ	Decreased absolute counts of CD4+ T cells and CD19+ B cells but not CD8+ T cells and CD56+ NK cells; no significant change in absolute Tregs counts
Campian et al. (2015) (40)	• Total: 10 • Median age (range): 55.5 (40–67) • Male: 8; female: 2 • Grade III: 3; grade IV: 7	Baseline, 6 weeks post-RT+TMZ	Decreased counts of CD4+, CD8+ T cells, and NK cells; no significant change in absolute Tregs counts
Campian et al. (2017) (41)	• Total: 20 • Male: 10; female: 10 • Median age (range): 56.5 (27–70) • Grade III: 6; grade IV: 14	Baseline, the end of RT+TMZ and 1, 3, 5, and 8 months post-RT+TMZ	Decreased absolute counts of Tregs, CD3+, CD4+, and CD8+ T cells, including naïve, central memory, and effector memory subsets
Pellegatta et al. (2018) (42)	• Total: 24 • Male: 16; female: 8 • Mean age: 54±10.7 • Grade IV: 24	Baseline, post-RT+TMZ	Decreased counts of CD4+, CD8+ T cells, and NK cells

RT, radiation therapy; NK, natural killer; NKT, natural killer T cells; TMZ, temozolomide; Tregs, regulatory T cells.

The total lymphocyte count (TLC) is classified as normal (≥1000 cells/mm^3^) or abnormal (<1000 cells/mm^3^), and grade 3/4 lymphopenia (<500 cells/mm^3^ for CD8+ T cells and <200 cells/mm^3^ for CD4+ T cells) is considered severe. Although the number of affected patients with grade 3/4 *versus* 1/2 lymphopenia varied significantly between studies, lymphopenia was among the most frequent hematologic adverse effects in patients with glioblastoma/gliomas during or after standard radio- and/or chemotherapy (46–54). A lower TLC before therapy predicts severe lymphopenia during treatment (46, 48, 55, 56). Furthermore, increasing age is an important factor that significantly contributes to the severity of therapy-induced lymphopenia; in the elderly group of patients (median age, 71 years), only 57% (out of n=72) had a normal baseline TLC (50) *versus* 97% (out of n=336) in the adult group (median age, 58 years) (57). Older age is independently associated with severe lymphopenia in patients with glioblastoma (55). Sex is another confounding factor of therapy-related lymphopenia. In two large cohort studies (n>700 and n>2000), women were found to be more likely to develop lymphopenia than men (29, 58). Remarkably, 21-47% of patients with glioblastoma/gliomas treated with radiochemotherapy developed long-lasting grade 3/4 lymphopenia (29, 47–52, 58–60), although there were studies reporting a less frequent occurrence of grade 3/4 lymphopenia during or after standard therapy (2.9-10%) (53, 61, 62). Moreover, patients with recurrent glioblastoma exhibited a significantly lower TLC compared with patients with newly diagnosed glioblastoma (63), and lymphopenia reached 76.5% for a standard temozolomide regimen in patients with recurrent glioblastoma (64). The post-treatment TLCs remained significantly lower than the baseline counts, even after 12 months, in adult (52) and elderly (50) patients with glioblastoma and in children with central nervous system tumors (65). Patients who developed severe CD4+ lymphopenia during standard therapy experienced higher hospitalization and infection rates (66, 67). However, the major cause of death in patients with grade 3/4 lymphopenia was tumor progression rather than infection (67).

It is widely accepted that lymphocytes are key effector cells in the immune response to tumors, and that anti-cancer T cell-mediated immunity is key to improving survival outcomes, although the contribution of other immune cell types should not be underestimated (68). Unsurprisingly, standard therapy-related grade 3/4 lymphopenia was associated with shorter overall survival in multivariate analyses (**Table 2**) (50, 56, 57, 60, 67) and meta-analysis (69). However, in Byun et al.’s study (57), severe lymphopenia was an independent prognostic factor only when the authors included patients with grade 3/4 lymphopenia at 3-month time of therapy but not when patients were included at any time point within 3 months of therapy. Similarly, Le Rhun et al. (29) reported that grade 3/4 lymphopenia during concomitant radiochemotherapy but not during maintenance temozolomide chemotherapy was significantly associated with inferior overall survival in univariate and multivariate analysis. Deng et al. (29) reported that lymphopenia during standard therapy was associated with overall survival in a multivariate analysis; however, no association was found between overall survival and lymphopenia at other time points (preoperative, pre-radiotherapy, or first recurrence). It may be concluded that the recovery rate from severe lymphopenia and the use of different time points to define treatment-related lymphopenia may be important modifiers of the prognostic power of TLC (57).

**Table 2:** Standard therapy-related grade 3/4 lymphopenia is a predictor of shorter overall survival in patients with glioblastoma/gliomas.

References	Patient characteristics	Baseline and post-therapy lymphocyte counts	Correlation with overall survival	Limitations/comments
Grossman et al. (2011) (67)	• Total: 96 • Mean age (range): 57.4 (28-85) • Male: 48 (50%); female 48 (50%) • Grade IV: 84%	• Baseline CD4 count range (median): 90-2010 (664) cells/mm^3^ • Patients with baseline CD4 >1000 cells/mm^3^: >90% • CD4 count range (median) at 2 months of therapy: 8-1580 (255) cells/mm^3^ • Patients with CD4 <200 cells/mm^3^ at 2 months of therapy: 40%	• CD4 <200 vs >200 cells/mm^3^: mOS 13.1 vs. 19.7 months, p=0.002 • HR 1.66, 95% CI 1.05-2.64, p=0.03 after adjustment for age, KPS, grade, and extent of surgery	• Prospective • 82% of patients were taking steroids • No data on *MGMT* promoter methylation status
Huang et al. (2015) (56)	• Total: 183 • Median age (range): 54 (21-82) • Male: 115 (63%); female: 68 (37%) • Grade III: 41 (22%); grade IV: 142 (78%)	• Baseline TLC range (median): 500-6400 (1200) cells/mm^3^ • Patients with TLC <500 cells/mm^3^ during therapy: 29%	• TLC <500 vs >500 cells/mm^3^: mOS 12.5 vs. 20.2 months; 2-year OS: 19% vs. 42%, p<0.001 • No multivariate analysis	• Retrospective • 70% of patients were taking dexamethasone • No data on *MGMT* promoter methylation status and IDH mutation status (patients diagnosed between 2007 and 2012)
Mendez et al. (2016) (50)	• Total: 72 • Median age (range): 71 (65-86) • Male: 34 (47%); female: 38 (53%) • Grade IV: 100%	• Baseline TLC range (median): 300-3200 (1100) cells/mm^3^ • Patients with baseline TLC >1000 cells/mm^3^: 57% • TLC range (median) at 2 months after therapy: 200-2200 (650) cells/mm^3^ • Patients with TLC <500 cells/mm^3^ at 2 months of therapy: 21%	• TLC <500 vs ≥500 cells/mm^3^: mOS 4.6 vs. 11.6 months, p=0.003 • HR 2.76, 95% CI 1.30-5.86; p=0.008 after adjustment for extent of surgery, *MGMT* methylation status, steroid use, and RT dose	• Retrospective • Relatively small sample size • 56% of patients were >70 years • 85% of patients were taking steroids • No data on IDH mutation status (patients diagnosed between 2000 and 2013)
Rahman et al. (2016) (60)	• Total: 196 • Median age (range): 59 (23-90)	• Patients with TLC <500 cells/mm^3^ during therapy: 47%	• TLC <500 vs >500 cells/mm^3^: crude OS 14.1 vs. 18.2 months, p=0.003 • HR 1.80, p=0.023 after adjustment for age, KPS, and *MGMT* methylation status	• Conference abstract • Retrospective • No data on steroid use • No data on IDH mutation status (patients diagnosed between 2006 and 2010)
Byun et al. (2019) (57)	• Total: 336 • Median age (range): 58 (16-79) • Male: 187 (55.7%); female: 149 (44.3%) • Grade IV: 100%	• Baseline TLC range (median): 300-3740 (1370) cells/mm^3^ • Patients with baseline TLC >1000 cells/mm^3^: 97% • TLC range (median) at 3 months after therapy: 170-3070 (1120) cells/mm^3^ • Patients with TLC <500 cells/mm^3^ within 3-months of therapy: 35.5%	• TLC <500 vs >500 cells/mm^3^: mOS 18.2 vs. 22.0 months, p=0.028 • HR 1.04, 95% CI 0.81-1.35, p=0.756 after adjustment for age, total resection, *IDH1* mutation, and *MGMT* methylation status	• Retrospective • No data on steroid use • 5.4% of patients had *IDH* mutation and 20.2% had unknown status (patients diagnosed between 2006 and 2017) • No correlation in multivariate analysis

TLC, total lymphocyte count; MGMT – O^6^-methylguanine-DNA-methyltransferase; mOS, median overall survival.

### 2.2 Radiation-induced lymphopenia is a prognostic factor for mortality in virtually all solid cancers

Lymphocytes are one of the most radiation sensitive cells in the body (70). T lymphocytes are more sensitive to radiation than neutrophils, monocytes, NK cells, dendritic cells, or macrophages (71, 72). Only 30-50% of the T cells were found viable 48-72 hours post-exposure *ex vivo* to a single dose of 2 Gy (73). Exposure to low single dose radiation (0.3-0.5 Gy) also decreased the number of peripheral blood T lymphocytes (73). It also should be noted that not only total lymphocyte counts but also lymphocyte diversity and activity are affected by radiation (72). For instance, helper T cells (CD4+) are more radiosensitive than cytotoxic T cells (CD8+) (72). A comprehensive discussion of the effects of radiation on various immune cell populations in humans and mice is presented elsewhere (72). 

A significant drop in the peripheral lymphocyte counts after extracorporeal irradiation of the circulating blood was observed in calves (74). Similarly, radiation given only to circulating blood within a dialysis unit in humans awaiting kidney transplants produced severe and long-lasting lymphopenia (75, 76). The standard radiotherapy regimen for glioblastoma (60 Gy delivered in 30 fractions to a partial brain field) may cause toxicity in up to 98% of circulating lymphocytes (77). The development of severe lymphopenia after radiation without concurrent chemotherapy or steroids use has been reported in patients with brain, head and neck, breast, pancreatic, esophageal, cervical, uterine, and lung cancer (52, 78). Notably, according to systematic reviews and meta-analyses, post-radiation lymphopenia is associated with shorter survival in pancreatic and lung cancer (79–81), head and neck and esophageal cancer (79, 82–84), urological cancer (85), nasopharyngeal and cervical cancer (47) and many other solid tumors (69, 78, 79, 86–88). In agreement, a meta-analysis including 16 cancer types supports that radiation-induced lymphopenia is a significant prognostic factor for mortality in virtually all solid cancers (79). Taken together, radiotherapy *per se* is a strong and primary inducer of lymphopenia regardless of the type of chemotherapy received by cancer patients.

### 2.3 Chemotherapy and corticosteroids may exacerbate radiation-induced lymphopenia

Temozolomide and dexamethasone are also lymphodepleting agents that may exacerbate radiation-induced lymphopenia in patients with glioblastoma/gliomas (43, 59, 89). In patients with grade II-III glioma (n=151), concurrent radiochemotherapy and the duration of adjuvant chemotherapy were significantly associated with lymphopenia in multivariate analysis (62). In a cohort of 39 patients with advanced metastatic neuroendocrine tumors who received temozolomide (no radiation, minimal use of concurrent steroids by 6/39 patients), the incidence of severe lymphopenia was 46% after 4 months and >60% after 6 months of temozolomide treatment, and persisted in at least 30% of patients during the 12 months following treatment discontinuation (90). The majority of patients with melanoma (>60-70%) developed lymphopenia after serial treatment cycles with different temozolomide dosing regimens and schedules (91–93). The development of lymphopenia by temozolomide can be caused by the negligible expression of *MGMT* and/or multidrug resistance proteins in peripheral blood lymphocytes. It should be noted that not only temozolomide may cause or promote long-term lymphopenia. For instance, in patients with primary breast cancer receiving other types of chemotherapy regimens without radiotherapy and steroids, CD4+ T cells remained significantly depleted even 9 months post-chemotherapy (reaching medians of only 60% of initial levels) (94).

### 2.4 Steroid-induced immunosuppression

Dexamethasone has comparatively low mineralocorticoid properties, high glucocorticoid potency, and a long biological half-life. However, corticosteroids exhibit adverse systemic effects, the most common of which are immunosuppression, hyperglycemia, hypertension, osteoporosis, myopathy, diabetes, and thromboembolic events (95, 96). The severity of these adverse effects is usually correlated with the total daily dose and duration of steroid application.

*In vitro* and *ex vivo* studies have shown that steroids alter the maturation of dendritic cells, inducing tolerogenic cells that express low levels of major histocompatibility complex, costimulatory molecules, and cytokines, resulting in hypo-responsiveness and an anergic state in naïve and memory T cells primed by such dendritic cells (97–100). Steroids impair T cell receptor signaling and the expression of many cytokines, chemokines, and adhesion molecules, affecting the development, polarization, activation, and migration of T cells, and promoting the formation of Tregs (97–100).

In chemoradiotherapy- and resection-naïve patients with glioblastoma, baseline lymphopenia is more frequently observed in dexamethasone-treated patients than in dexamethasone-naïve patients (28). In general, dexamethasone use is significantly associated with lower lymphocyte counts in patients with glioblastoma (43, 89). Dexamethasone use >2 *versus ≤*2 mg/day was independently associated with severe lymphopenia (3-month rate: 43.7% *versus* 19.8%; p<0.001) (101). To elucidate how short-term dexamethasone treatment affects the immune system of patients with glioblastoma, Gustafson et al. analyzed peripheral blood samples collected perioperatively and before radiochemotherapy (102). The CD4+ cell count was significantly lower in dexamethasone-treated patients than in dexamethasone-naïve patients (102). Changes in the expression of costimulatory and antigen-presenting molecules in peripheral monocytes were more pronounced in patients receiving dexamethasone (102). Patient-derived CD14+ monocytes had an immunosuppressive phenotype with defective direct T cell stimulation and dendritic cell differentiation capacity (102). In another study, dexamethasone treatment before glioblastoma resection affected the main immune cell populations, including CD4+ and CD8+ T cells, CD66b+ neutrophils, CD14+ monocytes, non-Vδ2 γδT cells, and NK cells (especially CD56high) (103). Similarly, analysis of pre-surgery blood samples revealed that patients with glioma (n=139) who received dexamethasone (45.3%) had the lowest B, NK, monocyte, CD4+, and CD8+ T cell, and total lymphocyte counts (104). Cook et al. examined peripheral blood samples from patients with malignant pleural mesothelioma who received 4 mg dexamethasone thrice prior to undergoing standard chemotherapy (105). The authors observed substantial immunomodulatory effects in response to dexamethasone administration, particularly affecting CD4+ and CD8+ T cells and dendritic cell subtypes. The proportion of Tregs did not change; however, a significant increase in their proliferation and activation was observed (105). In contrast, the absolute number of Tregs was significantly lower in dexamethasone-treated patients with glioblastoma than in normal controls or dexamethasone-untreated patients (102, 103).

Moreover, patients with glioblastoma receiving steroids had higher counts of blood MDSCs than those who did not receive steroids (35) or were on steroids for a shorter period (36). Dexamethasone has been also shown to induce MDSCs in preclinical transplantation models, and its dose positively correlated with the absolute number of MDSCs in transplant recipients (106). In addition, patients with glioblastoma receiving steroids had significantly higher neutrophil counts (58, 69, 89, 103, 104, 107). However, other research groups found no significant difference in neutrophil counts in patients receiving or not receiving steroid therapy (108) or revealed only a weak correlation between the dexamethasone dose and NLR (109). Finally, perioperative corticosteroid treatment impaired tumor-infiltrating dendritic cells in patients with newly diagnosed gliomas (110). Altogether, in accordance with *in vitro* and *ex vivo* studies, dexamethasone (without radiochemotherapy) adversely affects the immune system of patients with cancer.

### 2.5 Dexamethasone use is a prognostic factor for shorter survival

Steroid use as a negative prognostic indicator of survival in patients with glioblastoma/gliomas was recognized approximately three decades ago (111, 112) and confirmed by modern retrospective research (**Table 3**) (9, 55, 113–119). In congruence, a large cohort study (n=2002) (58) and meta-analysis (120) reported that steroid use was associated with worse overall survival. However, some studies have demonstrated no association between steroid use and outcomes in patients with newly diagnosed glioblastoma in univariate or multivariate analyses (50, 67, 107, 121–124). In some retrospective studies, steroid use was associated with worse overall survival in patients treated with radiotherapy only but not in those treated with both radiation and chemotherapy (9, 116), and *vice versa* (116). Nevertheless, when the patient cohorts were analyzed as a whole, dexamethasone use was associated with a negative prognosis (**Table 3**).

**Table 3:** The use of dexamethasone is a predictor of shorter overall survival in patients with glioblastoma/gliomas.

References	Patient characteristics	% of patients on corticosteroid therapy (Yes *versus* No)	Correlation with median overall survival in multivariate analysis	Limitations/comments
Gorlia et al. (2008) (9)	• Total: 573 • Median age (range): 56 (19-71)	71% vs. 29%	N=547; HR 1.36, 95% CI 1.11-1.67, p=0.003 after adjustment for age, sex, WHO performance status, extent of resection, MMSE score, and treatment assignment	• Retrospective • 64% of patients had unknown *MGMT* promoter methylation status • No data on IDH mutation status (patients diagnosed between 2000 and 2002)
Gorlia et al. (2012) (113)	• Total: 300 • Median age (range): 53.5 (18-78) • Male: 196 (65.3%); female: 104 (34.7%)	65.3% vs. 34.7%	N=189; HR 2.01, 95% CI 1.40-2.88, p=0.0001 after adjustment for WHO performance status, presence of neurological deficits, number of target lesions, tumor size, frontal tumor location, and prior chemotherapy with TMZ	• Retrospective • Recurrent patients were from eight multicenter phase I and II trials and received the heterogeneous treatments • No data on *MGMT* promoter methylation status • No data on IDH mutation status (patients diagnosed between 1999 and 2010)
Michaelsen et al. (2013) (114)	• Total: 225 • Median age (range): 59.2 (22.6-75.4) • Male: 145 (64.4%); female: 80 (35.6%)	73.3% vs. 25.3%	HR 2.06, 95% CI 1.47-2.87, p<0.0001 after adjustment for age and ECOG performance status	• Retrospective • 27.5% of patients had unknown *MGMT* promoter methylation status • No data on IDH mutation status (patients diagnosed between 2005 and 2010)
Tieu et al. (2015) (115)	• Total: 196 (derivation cohort) • Average age: 54 • Male: 125 (64%); female: 71 (36%)	95% vs. 5%	HR 1.04, 95% CI 1.01-1.07, p=0.02 after adjustment for age, ECOG performance status, and extent of resection	• Retrospective • No data on *MGMT* promoter methylation status • No data on IDH mutation status (patients diagnosed between 2004 and 2011) • A correlation between survival and time-weighted mean dexamethasone dose, calculated by regularly measuring dexamethasone dose from start of RT to 4 weeks following completion of RT, was analyzed
	• Total: 197 (validation cohort) • Average age: 54 • Male: 126 (64%); female: 71 (36%)	88% vs. 12%	HR 1.08, 95% CI 1.04-1.11, p<0.0001 after adjustment for age, ECOG performance status, and extent of resection	
Pitter et al. (2016) (116)	• Total: 622 • Mean age (range): 56.5 (19-71) • Male: 396 (63.7%); female: 226 (36.3%)	83.9% vs. 16.1%	12.9 vs. 20.6 months, p<0.0001; HR 1.512, 95% CI 1.2058-1.8960, p=0.00034 after adjustment for RTOG RPA Class and concurrent TMZ	• Retrospective
	• Total: 832 • Median age (range): 62 (19-86) • Male: 497 (59.7%); female: 335 (40.3%)	44.5% vs. 55.5%	12.1 vs. 15.7 months, p<0.001; HR 1.18, 95% CI 1.02-1.37, p=0.024 after adjustment for age, KPS, extent of resection, and treatment assignment	
	• Total: 573 • Mean age (range): 55.8 (18.6‐70.8)	71.2% vs. 28.6%	12 vs. 17 months, p<0.0001; HR 1.52, p=0.004 for the RT group; HR 1.2, p=0.2 for the RT+TMZ group after adjustment for age, extent of resection, and WHO performance status	
van Linde et al. (2017) (117)	• Total: 299 (recurrent) • Mean age (range): 57 (19-85) • Male: 202 (67.6%); female: 97 (32.4%)	61.5% vs. 34.8%	HR 1.85, 95% CI 1.33-2.59, p=0.001 after adjustment for age, sex, extent of resection, tumor extent, ECOG performance status, recurrence-free interval, and treatment assignment	• Retrospective • Patients received different systemic treatments at recurrence • No data on *MGMT* promoter methylation status • No data on IDH mutation status (patients diagnosed between 2005 and 2014)
Coleman et al. (2018) (118)	• Total: 100 • Median age (range): 48 (18-70) • Male: 69 (69%); female: 31 (31%)	63.7% vs. 36.3%	N=91; HR 1.84, 95% CI 1.05-3.24, p=0.034 after adjustment for ECOG performance status, NLR, and RMH score	• Retrospective • Relatively small sample size • 85% of patients had unknown *MGMT* promoter methylation status • 82% of patients had unknown IDH mutation status (patients diagnosed between 2004 and 2016)
Hui et al. (2019) (55)	• Total: 319 • Median age (range): 57 (21-82) • Male: 195 (61%); female: 124 (39%)	High-dose cohort (>2 mg/day, 47%) vs. low-dose cohort (≤2 mg/day, 53%)	N=231; HR 1.131, 95% CI 1.059-1.208, p<0.001 after adjustment for age, sex, extent of resection, *MGMT* methylation status, RT technique, and baseline ALC	• Retrospective • 28% of patients had unknown *MGMT* promoter methylation status • No data on IDH mutation status (patients diagnosed between 2007 and 2016)

ALC, absolute lymphocyte count; ECOG, the Eastern Cooperative Oncology Group; KPS, Karnofsky Performance Status; MGMT – O^6^-methylguanine-DNA-methyltransferase; MMSE, Mini-Mental State Examination; NLR, neutrophil-to-lymphocyte ratio; OS, overall survival; RMH, Royal Marsden Hospital prognostic score; RT, radiotherapy; RTOG RPA, Radiation Therapy Oncology Group Recursive Partitioning Analysis; TMZ, temozolomide; TTFields, tumor-treating alternating electric fields; WHO, World Health Organization.

In addition, multivariate analyses revealed that there were negative associations between baseline corticosteroid use and overall survival in patients with glioblastoma receiving checkpoint inhibitors (125, 126). In systematic reviews and meta-analyses, administration of steroids for supportive care (e.g., disease-related symptoms) or brain metastases was associated with significantly worse overall survival in non-small cell lung cancer and melanoma patients receiving immune checkpoint inhibitors (127, 128). In contrast, steroids used to mitigate adverse events did not negatively affect overall survival (128). However, steroid timing for immune-related adverse events may be associated with survival in patients receiving immune checkpoint inhibitor therapy. In large multicenter retrospective cohort study (n=20163), systemic steroids for immune-related adverse events were associated with significantly improved survival compared with those who received steroids for other reasons or no steroid treatment (mOS, 21.3 *versus* 13.6 *versus* 15.8 months; P<0.001) (129). However, among those who received steroids for immune-related adverse events, early steroid use (<2 months after immune checkpoint inhibitor initiation) was associated with reduced relative survival benefit in comparison to later steroid use, regardless of immune checkpoint inhibitor continuation or cessation following steroid initiation (mOS after immune checkpoint inhibitor cessation, 4.4 *versus* 16.0 months; mOS after immune checkpoint inhibitor continuation, 16.0 *versus* 29.2 months; P<0.001) (129).

### 2.6 Can corticosteroids compromise survival in glioblastoma?

Dexamethasone treatment significantly decreased survival in irradiated glioma-bearing mice in a genetically engineered mouse model of glioma (116) and increased proliferation, invasion, and angiogenesis in a human glioma stem cell-derived orthotopic tumor model (130). However, in a pediatric low-grade glioma cohort (n=191), no significant difference was observed in the short- or long-term tumor growth rates or in the progression-free survival of patients treated with or without perioperative dexamethasone, irrespective of gross total or incomplete resection (131). The negative correlation between dexamethasone use and overall survival reported in most large cohort studies (**Table 3**) might be due to less extensive surgery, more aggressive tumor growth, and edema in patients requiring steroid treatment (116, 132). A higher daily steroid dose (>2 mg/day) in comparison to a lower daily steroid dose (≤2 mg/day) was independently associated with median overall survival (n=319, 12.6 *versus* 17.9 months, p<0.001); however, in patients with gross total resection, the steroid dose was not prognostic for overall survival (55). Moreover, overall survival of patients receiving ≤4 mg/day dexamethasone did not differ significantly from that of patients who did not receive steroids (55). These data suggest that tumor size might still be a dominating confounding factor in the association between dexamethasone use and overall survival. However, in patients with recurrent glioblastoma receiving >4.1 *versus ≤*4.1 mg/day dexamethasone and treated with tumor-treating alternating electric fields (TTFields) (n=120), median overall survival was 4.8 and 11.0 months (p<0.0001), respectively, and tumor size did not differ statistically between patient cohorts (119). Furthermore, the analysis of three large independent cohorts (total n>2000) confirmed a negative association between dexamethasone use and overall survival after adjustment for age, extent of resection, and performance status (116). Overall, there can be confounding factors other than tumor size that modify the prognostic significance of steroid use. Steroid treatment-related systemic immunosuppression or other steroid-related adverse effects, such as the deregulation of blood glucose levels, may also affect patient survival. Hyperglycemia before surgery, during radiation, or radiochemotherapy was independently associated with poorer progression-free and overall survival in patients with glioblastoma after adjusting for the mean daily dexamethasone dose and other prognostic variables (115, 133–138). As each patient receives personalized dexamethasone treatment, it is not possible to prospectively determine how dexamethasone use, its daily dose, and the duration of treatment affect cancer patient survival.

### 2.7 Lymphopenia is significantly associated with response and survival outcomes in patients with advanced cancer on immune checkpoint inhibitor therapy

Patients with solid tumors treated with programmed cell death 1 (PD-1) checkpoint inhibitors (n=167) with absolute lymphocyte counts >2000 cells/μl at baseline had an increased risk of immune-related adverse events on multivariate analysis (139). At the same time, patients with lymphopenia at baseline and persistent lymphopenia at three months (largely radiation-related) had a shorter time to progression compared to those who had baseline lymphopenia but recovered with absolute lymphocyte counts >1000 cells/μl at 3 months (HR 2.76, p<0.05) (139). These data suggest that lymphopenia may dramatically reduce the incidence of immune-related adverse effects but is associated with an efficiency of immune checkpoint inhibitors. In support, pretreatment absolute lymphocyte count (<600 cells/μl) was significantly associated with response to PD-1 inhibitors in patients with head and neck squamous cell carcinomas (n=34) (140). In another study, lymphopenia (<1000 cells/μl) upon initiation of PD-1 inhibitor nivolumab was not associated with poorer survival in patients with head and neck squamous cell carcinomas (n=100), but persistent lymphopenia under nivolumab was associated with poorer overall survival in multivariate analysis (HR 3.96, 1.19-13.17, p=0.034) (141). Similarly, patients with metastatic melanoma (n=116) with a normal lymphocyte count at baseline but who developed lymphopenia during immune checkpoint inhibitor therapy had significantly shorter progression-free survival (13.3 *versus* 16.9 months, p=0.025) and overall survival (28.1 *versus* 36.8 months, p=0.01) compared with patients who did not develop (142). In a prospective, observational study of patients with advanced non-small cell lung cancer (n=123) treated with immune checkpoint inhibitors, high absolute lymphocyte count (>1.01×10^9^/L) and absence of liver metastases were significantly associated with a durable clinical benefit defined as progression-free survival >6 months (143). Finally, a meta-analysis of patients with lung cancer who were treated with immune checkpoint inhibitors confirms that lymphopenia is associated with poor survival (144).

## 3 Implications of standard therapy-related immunosuppression for immunotherapy or oncolytic virotherapy clinical trials

### 3.1 Standard therapy-related immunosuppression is a barrier to immunotherapies

It is rationalized that standard radio/chemotherapy is important for reducing tumor mass and increasing neoantigenic tumor load, and that it can enhance the immunogenicity of the tumor microenvironment by inducing immunogenic cell death, which promotes anti-tumor immunity (145). On the other hand, radiotherapy induces systemic and intratumoral lymphopenia (146). Furthermore, glioblastoma cells are intrinsically resistant or acquire resistance to chemotherapy, which may promote aggressiveness and enhance their immunosuppressive properties (101, 147–149). Temozolomide is not a potent inducer of *bona fide* immunogenic cell death in human glioblastoma cells (150–152) and is not included in the shortlist of anti-cancer chemotherapeutics with the capacity to consistently induce immunogenic cell death (153). Although ionizing radiation can induce immunogenic cell death, it is critical to realize that conventional radiation treatment trigger a combination of cell death processes, such as apoptosis, necrosis, autophagy, mitotic catastrophe, and senescence, eliciting both immune-activating and suppressing responses (154). Additionally, it is widely recognized that immunogenic cell death cannot drive anti-cancer immunity in the presence of general immunological defects. A growing list of intrinsic and acquired immune-related resistance mechanisms that limit the efficient exploitation of immunogenic cell death in cancer immunotherapy has emerged (153, 155, 156). In most studies, comparative analyses of newly diagnosed and recurrent (matched or unmatched) glioblastoma samples revealed no significant differences in the density of immune cell subsets or exhaustion profiles of tumor-infiltrating T cells (157–165). On the contrary, monocyte-derived tumor-associated macrophages are incessantly recruited in recurrent glioblastoma, and the macrophage/microglia ratio is increased in both the central and marginal areas of the recurrent tumors in comparison to newly diagnosed glioblastoma (159, 166). The lack of significant changes in the immune infiltration profiles of cytotoxic lymphocytes in recurrent glioblastoma questions the putative positive immunomodulatory role of standard radiochemotherapy within the tumor microenvironment.

Furthermore, T cell receptor sequencing demonstrated a contracted T cell receptor repertoire diversity concomitant with an increased frequency of activated memory T cells among tumor-infiltrating lymphocytes in patients with recurrent glioblastoma after standard therapy (63). The youngest naïve T cells are continuously exported from the thymus as “recent thymic emigrants” and maintain T cell diversity in the periphery with a particularly important contribution in adults recovering from lymphopenia (167, 168). There is emerging evidence that thymic emigrants can mount robust immune responses (169). CD8+ thymic emigrants have been found to account for the majority of tumor antigen-binding cells in peripheral blood mononuclear cells in patients with glioblastoma (170). In patients vaccinated with autologous tumor lysate-pulsed dendritic cells, the levels of expanding CD8+ recent thymic emigrants strongly correlated with vaccine-elicited cytokine responses and predicted survival outcomes (170). As the thymus significantly degenerates with age (thymus involution) (65, 171), in adult and elderly patients, the restoration of heterogeneous populations of T cells and re-establishment of T cell immunocompetence after standard therapy is a slow and frequently incomplete process, which may progress primarily through thymic-independent peripheral expansion of the remaining mature T cell populations with reduced T cell receptor repertoire diversity (63, 172). Therapy-related contraction in the T cell receptor repertoire diversity may potently decrease the chances of a successful anti-tumor response.

Since immunotherapeutics are tested in patients with glioblastoma concurrently with or after standard therapy, standard therapy-related immunosuppression presents a barrier to the success of immunotherapies (86). It has been repeatedly reported that systemic chemotherapy (173–177) or CD4+/CD8+ T cell depletion (150, 178–186) abrogates the survival benefit of immunotherapies or oncolytic virotherapy in preclinical models. Therefore, it appears counterintuitive to combine lymphotoxic radiotherapy/chemotherapy with immunotherapy or virotherapy. Pellegatta et al. reported that standard therapy significantly decreased CD8+, CD4+, and NK cell counts in patients with glioblastoma (n=24), and the administration of adjuvant temozolomide had a negative effect on the increase of the CD8+ T cell subset and the generation of CD8+ T cell-associated anti-tumor memory elicited by dendritic cell vaccination (42). A negative effect on anti-tumor immunity of temozolomide administration as an adjuvant to dendritic cell vaccination in patients with recurrent glioblastoma was also observed in another clinical study (187). It is also noteworthy that in patients with newly diagnosed *MGMT*-unmethylated glioblastoma, personalized neoantigen peptide vaccines induced circulating neoantigen-specific T cell responses and a significant increase in infiltrating CD8+ T cells at relapse only in those who did not receive dexamethasone during vaccine priming (188). Conversely, the potential positive interaction of radio/chemotherapy with immunotherapy or oncolytic virotherapy was also demonstrated in many immunocompetent rodent models, and the rationale for their combination and possible limitations are intensively debated in the literature. A few studies have argued that immunosuppression does not prevent immune responses induced by a vaccine in patients with glioblastoma (189) and have pointed to a putative positive immunomodulatory role of temozolomide in dendritic cell vaccination or multimodal immunotherapy (190, 191). However, no large, well-designed randomized controlled trials have been conducted to prove the positive effects of the addition of immunotherapy or oncolytic virotherapy to standard therapy, and only minor subsets of patients have benefited from conducted clinical trials (3–7).

### 3.2 The need for shifting the treatment paradigm

An increasing number of studies have reported an association between the radiotherapy dose/volume and lymphopenia in glioblastoma (56, 77, 192, 193). The severity of radiation-induced lymphopenia depends on the technique of radiotherapy, fraction number (fractionation regimen), dose per fraction (irradiation dosage), field size, and other variables (78, 194–197).

Patients with glioblastoma treated with moderately hypofractionated radiotherapy (58.5 Gy in 25 fractions, n=78) had a significantly reduced rate of grade ≥2 lymphopenia at 6 months post-radiotherapy in comparison with conventionally fractionated radiotherapy (60 Gy in 30 fractions, n=145), with no difference in overall survival between groups (27.2 *versus* 26.6 months) (198). In addition, in patients with pancreatic cancer, hypofractionated radiotherapy in comparison to standard radiotherapy also considerably reduced the risk and severity of radiation-induced lymphopenia (78, 199). In a study of patients with glioblastoma aged ≥60 years (n=100), there was no difference in survival between those receiving standard radiotherapy (60 Gy in 30 fractions over 6 weeks) or short-course radiotherapy (40 Gy in 15 fractions over 3 weeks) (200). In congruence, a meta-analysis of controlled trials testing the impact of radiotherapy hypofractionation on the survival of patients with glioblastoma reported comparable survival outcomes between hypofractionation and standard radiotherapy (201).

Patients with glioblastoma receiving intensity-modulated radiotherapy (IMRT, n=150) had a significantly decreased incidence of severe lymphopenia (20% *versus* 37%; p=0.005) compared with patients treated with three-dimensional conformal radiotherapy (3D-CRT, n=186) (57). Compared with standard-field radiotherapy (n=164), limited-field radiotherapy (n=46) was associated with less grade 3/4 lymphopenia after radiochemotherapy, though not statistically significant (15.5% *versus* 33.8%; p=0.12), and did not adversely affect progression-free and overall survival in patients with glioblastoma (193). In a prospective, randomized phase II glioblastoma trial, a comparison of the Radiation Therapy Oncology Group (RTOG) and The University of Texas MD Anderson Cancer Center (MDACC) radiation treatment guidelines revealed that overall survival was superior in the MDACC group (17 *versus* 12 months, p=0.015, n=25 per arm) (192). In locally advanced pancreatic cancer, 13.8% and 13.6% of patients in the stereotactic body radiation therapy group *versus* 71.7% and 46.0% of patients in the conventional chemoradiation therapy group had severe lymphopenia at 1 month and 2 months, respectively, after starting radiotherapy (202).

In a randomized phase II glioblastoma trial of proton *versus* X-ray (photon) therapy with concurrent temozolomide, the rates of grade 3/4 lymphopenia occurrence were lower for proton therapy (4/28, 14%) than for X-ray (photon) therapy (22/56, 39%, p=0.024) (203). Grade 3/4 lymphopenia was significantly associated with baseline absolute lymphocyte counts, whole-brain mean dose, and brain volumes receiving 5–40 Gy (203). Similarly, in esophageal cancer patients undergoing neoadjuvant chemoradiotherapy, a greater proportion of patients in the intensity modulated radiation therapy group (55/136, 40.4%) developed grade 4 lymphopenia compared with patients in the proton therapy group (24/136, 17.6%, P<0.0001) (204). On multivariate analysis, proton therapy was significantly associated with a reduction in grade 4 lymphopenia risk (204). In a retrospective nonrandomized study of the comparative effectiveness of proton (n=391) and photon (n=1092) therapy as part of concurrent chemoradiotherapy for locally advanced cancer, proton chemoradiotherapy was associated with significantly reduced acute adverse events but with similar disease-free and overall survival (205). Numerous comparative studies, including prospective and/or randomized, support the hypothesis that proton therapy is associated with improved toxicity and results in outcomes at least equivalent to those of photon therapy (206, 207). Results from the large randomized NRG BN001 (NCT02179086) phase II trial comparing dose-escalated photon IMRT or proton beam radiation therapy *versus* standard-dose radiation therapy and temozolomide in treating patients with newly diagnosed glioblastoma are awaited. Moreover, three phase III trials directly comparing proton therapy to photon therapy across a broad variety of malignancies are currently accruing (206).

Taken together, although all these studies should be currently considered as hypothesis-generating rather than conclusive, and require multi-center validation in larger cohorts of patients, they clearly suggest that modifications in the standard radiation treatment paradigm might reduce negative effects on the immune system without compromising overall survival, potentially strengthening the therapeutic efficacy of prospective immunotherapies (208).

Temozolomide exerts a negligible therapeutic effect in patients with glioblastoma with an unmethylated *MGMT* promoter (209) and has been omitted from first-line therapy in some clinical trials (87). Temozolomide therapy could be excluded for patients with unmethylated *MGMT* promoter in immunotherapy/oncolytic virotherapy trials. The administration of immunotherapeutics as neoadjuvants (210–212) and local administration of therapeutics to the brain should also be considered (213).

Immunotherapy trials generally restrict steroid use at enrollment. Interestingly, the proportion of patients with glioma who were steroid-free at the end of chemoradiotherapy varied significantly in different studies (e.g., 78%, 55.5%, 28.6%, 27%, and 16%) (214). There is no standard treatment regimen for the steroid dexamethasone in neuro-oncology (215), which means that the choice of daily dose, duration of treatment, and tapering schemes are adapted for each patient. Body weight or patient age were not considered a modifier of the daily dose (96). It is not recommended to use high doses of dexamethasone for prophylaxis in asymptomatic patients; instead, the lowest dose must be considered to balance the desired effect and multiple adverse effects (216, 217). To avoid toxicity associated with prolonged exposure, dexamethasone should be tapered after achieving maximum clinical benefit with decrements of the previous dose until the lowest dose needed to maintain optimum neurological function is reached. Since it is important to control steroid use in patients with gliomas, the Response Assessment in Neuro-Oncology (RANO) Working Group developed consensus recommendations on steroid use endpoints in clinical trials in both adults and children with brain tumors (218). Nevertheless, based on accumulating clinical evidence, a less toxic and comparably effective alternative to dexamethasone is urgently required to treat edema in patients with brain tumors.

The search for methods to improve lymphocyte count recovery following prolonged lymphopenia is another important direction for clinical research (88). In patients with lymphopenia before standard therapy, T cells are sequestered in the bone marrow owing to decreased levels of sphingosine-1-phosphate receptor 1 (S1PR1) on the surface of T cells (28). Pharmacological stabilization of S1PR1 on the T cell surface has been suggested as a potential strategy to ameliorate bone marrow T cell sequestration and reverse baseline lymphopenia (28). However, S1PR1 plays a role in both adaptive and innate immune responses by regulating the recruitment, trafficking, and function of T cells and most innate immune cells (219). Moreover, S1PRs are expressed in glioblastoma and sphingosine-1-phosphate signaling is active in glioblastoma cells, contributing to the pathobiology of brain tumors (220). It is likely that modulators that stabilize S1PR1 might exert pleiotropic effects, including potentially adverse pro-tumorigenic effects.

In patients with glioblastoma and lymphopenia after standard therapy, neither the total lymphocyte count nor CD4+ cell recovery was augmented by the reinfusion of autologous lymphocytes harvested using apheresis prior to therapy (40). A recent prospective correlative study of patients with glioblastoma after radiochemotherapy (n=20) suggested that the expansion of circulating MDSCs due to increased myelopoiesis in the bone marrow may be associated with lymphopenia, which was supported by preclinical *in vivo* data (221). Moreover, in patients with glioblastoma and lymphopenia, a compensatory increase in the concentration of interleukin-7 (IL-7) and interleukin-15 (IL-15) was not observed (39). IL-7 and IL-15 are essential for homeostasis of circulating T lymphocytes (88). In a prospective cohort of patients with hepatocellular carcinoma (n=98), post-radiotherapy IL-7 levels were significantly positively correlated with total lymphocyte counts at 2 months (222). hIL-7-hyFc is a homodimeric IL-7, fused to the hybridizing IgD/IgG4 immunoglobulin domain. hIL-7-hyFc was well-tolerated and increased absolute lymphocyte counts in healthy humans after a single administration (223). In patients with recurrent glioblastoma (n=18), treatment with rhIL-7-hyFc restored and maintained total lymphocyte counts without serious toxicity and irrespective of steroid use during treatment with salvage therapies, such as temozolomide and/or bevacizumab (224). A large randomized controlled trial is required to validate the clinical benefits of rIhL-7-hyFc treatment in cancer. In general, based on available clinical data for the treatment of severe lymphopenia in cancer and non-cancer patients, rhIL-7 is a potent therapeutic candidate for immune reconstitution (88).

### 3.3 Detailed blood and/or tumor immunophenotyping may be valuable for immunotherapy and oncolytic virotherapy trials

In dendritic cell vaccinated patients with glioblastoma, CD8+ T cells levels (170, 225), the maximum count of CD3+*/*CD4+ T cells (226), the increased vaccination/baseline ratio of NK cells (227) and fold change in Tregs frequency (228) in peripheral blood, post-vaccination IFN-γ T cell responsiveness (225, 229, 230), higher tumor-infiltrating lymphocyte density (231), a low PD-1+/CD8+ ratio in tumor tissue (232), a low B7-H4 expression level in tumor tissue (233), a low cytotoxic T-lymphocyte-associated antigen 4 (CTLA-4) expression level on T cells (228), and a low programmed cell death 1 ligand 1 (PD-L1) expression level on myeloid cells (234) predicted therapy responses and/or were correlated with overall survival. In a phase II trial of a whole-cell lysate dendritic cell vaccine combined with standard therapy for newly diagnosed glioblastoma, patients with a low PD-1+/CD8+ ratio in tumor tissue had a median overall survival of 61 *versus* 20.7 months for patients with a high PD-1+/CD8+ ratio (232). Similarly, in a phase II trial of autologous heat shock protein peptide vaccine combined with standard therapy in newly diagnosed glioblastoma, a median overall survival in patients with low PD-L1 expression on myeloid cells was 44.7 *versus* 18.0 months for patients with high PD-L1 expression (234). There have also been attempts to identify responders by hierarchical clustering of a set of post-vaccine lymphocyte functional parameters (235). However, no prospectively validated robust predictive biomarkers for immunotherapy in patients with glioblastoma have been established.

The correlation between immune cell subsets, signatures, or markers and response to immunotherapy/oncolytic virotherapy and overall survival should be monitored in clinical trials to identify new or validate the proposed immunological-based predictive and prognostic variables. The first prospective, explorative, and observational IMMO-GLIO-01 trial (NCT02022384) was launched to examine the immune status of approximately 50 patients with glioblastoma or anaplastic astrocytoma during standard therapy. As stated in the goals of the study, it would be useful to estimate individual responses, stratify patients, and find suitable time points for the inclusion of additional immunotherapy (236). Since only 21% of patients with recurrent glioblastoma treated with the recombinant oncolytic poliovirus PVSRIPO experienced long-term survival (>3 years), and 79% of patients did not respond (237), researchers developed a robust method for cellular immunome monitoring to identify biomarkers that predict the response to virotherapy (238). This method is exploited in a phase II study in patients with recurrent glioblastoma (NCT02986178) to assess baseline and therapy-mediated changes in local and peripheral cellular immunomes.

## 4 Conclusion

**Figure 1:**
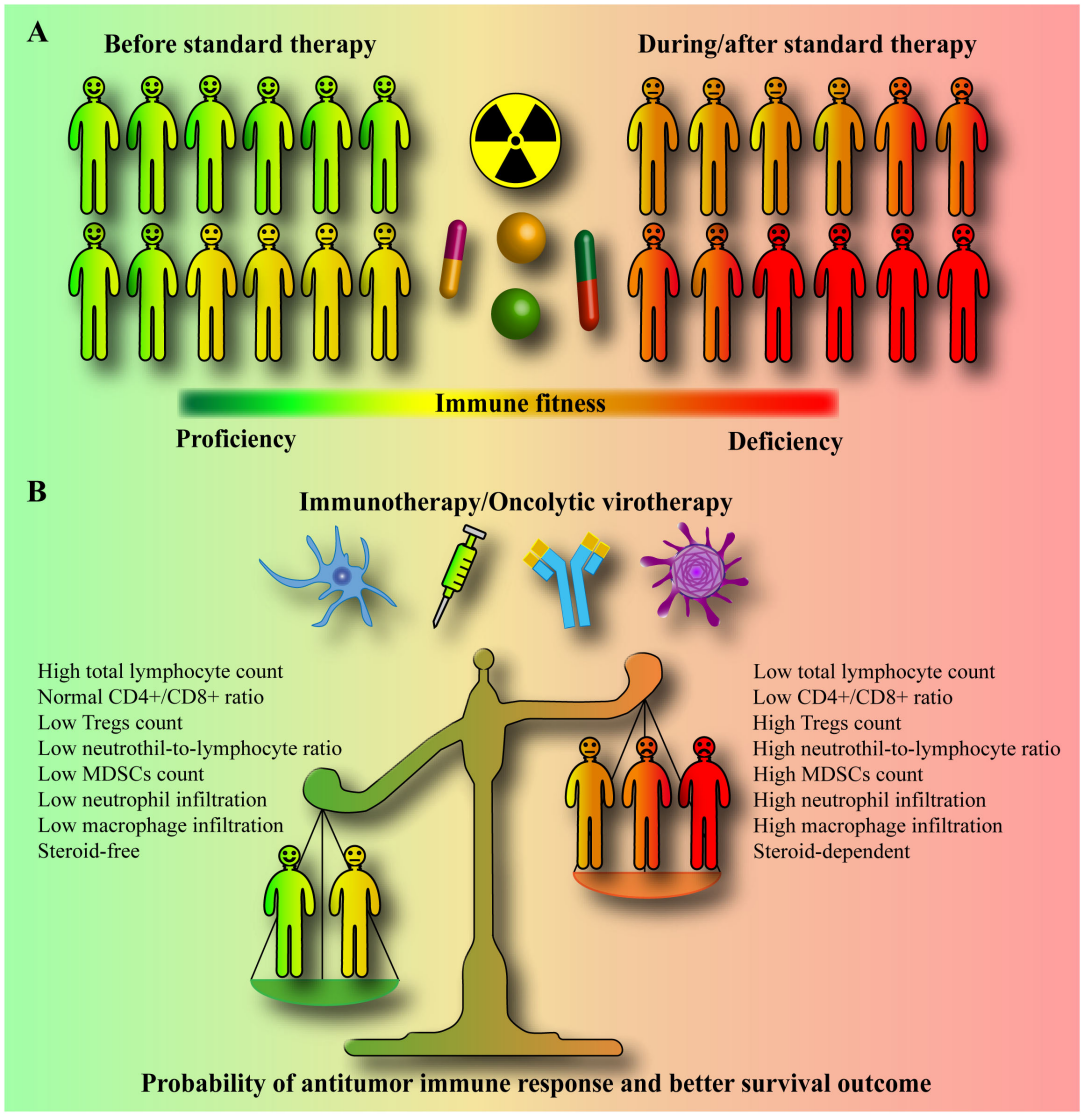
Standard therapy-promoted systemic immunosuppression may compromise the efficacy of immunotherapy/oncolytic virotherapy. **(A)** Prior to initiation of standard therapy, patients with glioblastoma/gliomas display varying degrees of immunosuppression. However, standard therapy, which includes radiation, a genotoxic drug temozolomide, and steroid dexamethasone, is a major cause of immune deficiency in patients, inducing long-lasting severe systemic immunosuppression and lymphopenia with a poor survival prognosis. The standard therapy differentially affects the immune system of each patient, with some patients developing moderate and some severe immunosuppression. Moreover, the rate and extent of lymphocyte count recovery after standard therapy also differ significantly between patients. **(B)** Immunotherapy and oncolytic virotherapy rely on the activity of the host’s own immune cells. The circulating and tumor-infiltrating CD4+, CD8+, NK, NKT, neutrophil, macrophage, myeloid-derived suppressor cell (MDSC), and regulatory T cell (Treg) counts, and subsets, and their relative ratios determine the immunological fitness of a patient. In clinical trials, immunotherapy and oncolytic virotherapy are largely tested in patients treated concurrently with or after standard therapy (in progressive/recurrent patients). Standard therapy-promoted immunosuppression/lymphopenia may limit the ability of the immune system to target glioblastoma. It is very likely that patients with lower systemic immune suppression may generally benefit from immunotherapy or oncolytic virotherapy much better than severely immune compromised patients who might be non-responsive to any extent. A high baseline neutrophil to lymphocyte ratio (NLR), a low post-treatment total lymphocyte count (TLC), and dexamethasone use are significant prognostic factors for shorter overall survival.

Delivering the right combinations with the right dosages at the right place during the right time is a difficult task in cancer treatment but may be highly relevant to immunotherapeutics. Although the tumor itself is immunosuppressive, clinical data provide compelling evidence that standard therapy exacerbates immune deficiency in patients with glioblastoma by promoting lymphopenia and systemic immunosuppression (**Figure 1A**). Prior to the initiation of standard therapy, patients with glioblastoma/gliomas display varying degrees of immunosuppression but rarely have severe lymphopenia. In contrast, clinical data indicate that standard therapy affects diverse immune cell subsets, with primary immunodeficiency related to long-lasting T cell lymphopenia. However, standard therapy differentially affects the immune system of each patient, and the rate and extent of lymphocyte count recovery after standard therapy differ significantly between patients. Most patients do not recover to baseline levels.

Standard therapy-promoted systemic immunosuppression and lymphopenia may limit the immune system’s ability to target glioblastoma (**Figure 1B**). Hence, changes in the standard therapy paradigm are required to increase the success of immunotherapies. Eligibility criteria, particularly relevant in neuro-oncology, and inadequate phase II study designs have been critically reviewed elsewhere (239). As for the design of clinical trials for glioblastoma to improve the effectiveness of immunotherapy/oncolytic virotherapy in general, the following aspects, supported by clinical data, must be taken into account (196, 197, 240, 241). First, the circulating blood within the blood vessels is recognized as an organ at risk for radiotherapy. Photon therapy, larger planning target volume, and higher brain dose were associated with increased risk of severe lymphopenia in glioblastoma, which correlates with poor survival. Therefore, the best efforts should be aimed to find and implement an efficient lymphocyte-sparing radiation modality (regimen and technique). Irradiation to the minimum necessary target using high-precision imaging (a minimal target definition), reduction of low-dose irradiation around the target (e.g., proton therapy), and a smaller number of fractions (i.e., hypo-fractionation) would be the strategy. Second, concurrent temozolomide and overall corticosteroid exposure during radiotherapy are contributing factors to lymphopenia. For trial enrollment, particular consideration should be given to patients without grade 2-4 lymphopenia, who are not expected to require steroids, and with an unmethylated *MGMT* promoter, for whom lymphotoxic temozolomide have to be completely omitted. Third, absolute/total lymphocyte counts should be monitored after radiotherapy, and appropriate effective interventions overcoming radiation-induced lymphopenia should be established and applied. Fourth, high NLR (31–33) and low post-treatment TLC are significant prognostic factors for shorter survival in patients with glioblastoma/gliomas. These and other cost-effective, widely and easily available clinically relevant prognostic immune variables associated with systemic inflammation and adaptive immunity (e.g., platelet-to-lymphocyte ratio (PLR), lymphocyte-to-monocyte ratio (LMR), systemic immune-inflammation index (SII), systemic immune response index (SIRI), or combinations thereof) (33, 242–248) should be reported and correlated with response and survival in immunotherapy/oncolytic virotherapy studies. In addition, since the use of different time points to define treatment-related lymphopenia has been reported to modify the prognostic power of lymphocyte counts, uniform time-points in standard therapy-related lymphopenia assessment should be established. Finally, detailed immunophenotyping of blood and/or tumor samples to assess the predictive/prognostic clinical significance of immune-related variables may also be valuable for future therapy decision-making in immunotherapy/oncolytic virotherapy trials.

## Author contributions

AAS: Conceptualization, Funding acquisition, Investigation, Visualization, Writing – original draft, Writing – review & editing. AOS: Writing – review & editing. MPV: Writing – review & editing. AAC: Writing – review & editing. OVA: Writing – review & editing. VAN: Writing – review & editing. VPC: Writing – review & editing.
